# The Bureau of Animal Industry

**Published:** 1900-04

**Authors:** Edgar W. Powell

**Affiliations:** Student, University of Pennsylvania, Veterinary Department


### THE BUREAU OF ANIMAL INDUSTRY.

By Edgar W. Powell,
STUDENT, UNIVERSITY OF PENNSYLVANIA, VETERINARY DEPARTMENT.

Perhaps it will be interesting as well as instructive for us to know something about the work done by this important division of the Department of Agriculture. Dr. D. E. Salmon is chief of the bureau. The Bureau of Animal Industry is divided up into several divisions, and covers a great many different kinds of work. Under the inspection division we have meat-inspection, inspection of vessels and export animals, Southern cattle inspection, prevention of scabies in sheep, inspection of export animals and dairy products, etc.

Then we have the pathological division, under which comes the black-leg investigation, Texas fever, glanders, rabies, infectious leukæmia in fowls, and other diseases of fowls. Under this head comes a good deal of miscellaneous work in the way of examining and determining specimens sent from all parts of the country and covering nearly all the pathological conditions to which our animals fall heir. Each of these specimens requires much laboratory work, sometimes a week being devoted to a single specimen. In each case a careful record is made and a complete answer sent to the sender.

Bacterial cultures are made also.

Under the biochemic division comes the preparation of tuberculin, mallein, serum for hog-cholera and swine-plague, as well as ink for stamping meat, etc.

To return to the branch or division of inspection. During the last year the inspection of cattle, sheep, calves, and swine was carried on at 138 abattoirs and packing houses in forty-one cities. In twelve of these cities the ante-mortem inspection was made in the stock-yards. The inspection of horses was conducted at one abattoir only. Many of the animals are rejected at the stock yards on account of pregnancy. These animals are allowed to be shipped out when desired for feeding or dairy purposes.

The total number of ante-mortem inspections in the year closing June, 1899, was 53,223,176. The number rejected upon this examination was 156,539. The growth of this feature of the work is shown by the fact that in 1892 the total number of inspections was only 3,889,459.

The total number of post-mortem inspections in the last year was 34,163,155. The cost of this inspection was $465,709.23. Of over two million hogs inspected microscopically only 1.87 per cent. contained living trichinæ. The cost of this work was 8.9 cents for each carcass examined.

All this work was done to prevent the spread of disease among the animals of the United States; to protect the consumer from diseased meat; to secure the admission and transmission of our animal products to foreign markets in good condition, and to maintain the reputation of the products at home and abroad.

It is reported that the annual loss of cattle from the disease known as black leg or symptomatic anthrax in the districts principally affected has ranged from 5 to 35 per cent. The Bureau of Animal Industry has for some time distributed a vaccine for the prevention of this disease, and this it is estimated has reduced the loss to 0.54 per cent. among the animals treated. During the last year 545,289 doses were distributed. The indications are that the contagion gradually dies out where systematic inoculations are carried out, and it was with the hope of eradicating the disease from many sections that the preparation of the vaccine was undertaken.

*Texas Fever Investigations.* The experiments of the Bureau demonstrated that Northern cattle might be made immune from Texas fever by inoculating them with the blood of immune animals. This has recently been adopted and practised with most satisfactory results by some of the experiment stations. The practical application of this discovery is of great importance both to the breeder of improved stock in the more Northern States and to the cattle raisers of the infected districts, as it permits of the improvement of Southern herds without the discouraging losses that have heretofore always occurred.

Experiments have been continued with a view of obtaining a mixture in which the cattle from the Texas fever districts may be dipped for the destruction of the ticks, and which at the same time will not injure the cattle. The effort has not been entirely successful, but the progress of the work leads to the hope that such a mixture may be produced. The difficulty of finding such a mixture is plain to those who know how tenacious of life is the tick, which is the carrier of the disease.

Investigations in Porto Rico show that the cattle-tick is prevalent there, but the ticks which were brought from there and placed upon cattle in the United States failed to produce Texas fever. Whether this result was accidental, or whether these ticks are without infectious properties, is of great importance. If further investigations show that the Porto Rican tick is free from the pyrosoma, the true contagion of the disease, and that the cattle of Porto Rica are susceptible, the introduction of a single animal bearing the pyrosoma might convert these comparatively harmless parasites into the most deadly scourge of the bovine race. This subject will be further experimented upon in the present year.

*Hog-cholera.* The preparation of serum for treating hog-cholera and swine-plague has been on a much larger scale than last year. The diseased herds in four counties in Iowa have been under treatment, the result showing a saving of 75 to 80 per cent. of the animals injected. It is evident that this mode of treatment is far in advance of anything tried heretofore.

*Sheep-scab.* For many years the scab has been prevalent among flocks, especially of the Western States and Territories, and sheep have been shipped from one State to another in violation of the law until the stock yards and cars have been almost continually infected. The result of this is that sheep could not be bought from any market without danger of bringing to the farm the contagion. It is one of the greatest evils the sheep industry has to contend with, not only damaging and often destroying the fleece, but so reduces the strength and condition of the animals that they often die or succumb to some other influence. The first step taken by the Department was to issue a pamphlet notifying transportation companies and shippers of the existence of the contagion, and pointing out the prohibition of shipment and the penalties provided by law. Subsequent to this an order was issued that diseased sheep discovered by inspectors in the channels of interstate commerce should be detained and dipped before going to their destination; also sheep purchased in infected yards for feeding should be dipped before they were allowed to go to the farms. It was found that some of the dips used by the stock yards were not efficacious, and that others were so severe or poisonous as to be dangerous. An order has consequently been issued specifying the kinds of dips to be used and the manner in which these should be prepared and applied. The effect of this has been satisfactory, and the number of diseased flocks received at the principal stock yards has been greatly diminished.

*Glanders.* Mallein is furnished only to local authorities for official use. There has been enough sent out in the year to test 5200 animals. Four cases of glanders were reported in the District of Columbia.

Dairy inspection or the inspection of dairy products is a very important part of the work of the Department, but I will not discuss it here.

The Department in May last sent the chief of the biochemic division to Berlin to attend the International Tuberculosis Congress, and over two hundred prominent men were at this congress as delegates from twenty-five nations. Papers by Virchow and Bollinger and others emphasized the dangers of meat and milk from tuberculous animals. It was the fortune of the chief of the biochemic division to meet at that congress, and afterward in visiting the chief and most important antitoxic serum laboratories and stations in Europe, the most important workers in biochemic lines. He found the scientific work of the Bureau of Animal Industry to be held in high regard by investigators in similar lines in the different European States. It is a great advantage to have the employés of the Bureau meet personally the scientific investigators in similar lines, thus affording an opportunity of exchange of ideas and the planning of coöperative work.

Experiments are in progress by the Department to produce an antitoxic serum for the prevention and treatment of tuberculosis.

